# Paternal HLA-C and Maternal Killer-Cell Immunoglobulin-Like Receptor Genotypes in the Development of Autism

**DOI:** 10.3389/fped.2016.00076

**Published:** 2016-07-28

**Authors:** Moriya Gamliel, Karen L. Anderson, Richard P. Ebstein, Nurit Yirmiya, David Mankuta

**Affiliations:** ^1^Department of Immunology and Cancer Research, Hadassah-Hebrew University Medical Center, Jerusalem, Israel; ^2^Department of Obstetrics and Gynecology, Hadassah-Hebrew University Medical Center, Jerusalem, Israel; ^3^Department of Psychology, National University of Singapore, Singapore; ^4^Department of Psychology, The Hebrew University of Jerusalem, Jerusalem, Israel

**Keywords:** autism, pregnancy, natural killer, KIR, HLA

## Abstract

Killer-cell immunoglobulin-like receptors (KIRs) are a family of cell surface proteins found on natural killer cells, which are components of the innate immune system. KIRs recognize MHC class I proteins, mainly HLA-C and are further divided into two groups: short-tailed 2/3DS activating receptors and long-tailed 2/3DL inhibitory receptors. Based on the Barker Hypothesis, the origins of illness can be traced back to embryonic development in the uterus, and since KIR:HLA interaction figures prominently in the maternal–fetal interface, we investigated whether specific KIR:HLA combinations may be found in autism spectrum disorders (ASD) children compared with their healthy parents. This study enrolled 49 ASD children from different Israeli families, and their healthy parents. Among the parents, a higher frequency of HLA-C2 allotypes was found in the fathers, while its corresponding ligand 2DS1 was found in higher percentage in the maternal group. However, such skewing in KIR:HLA frequencies did not appear in the ASD children. Additionally, analysis of “overall activation” indicated higher activation in maternal than in paternal cohorts.

## Introduction

Autism spectrum disorders (ASD) encompass a range of neurodevelopmental syndromes defined by difficulties in social communication and stereotyped behaviors (1). Recent data indicate a prevalence of up to 1 in 66 children (2). Although studies in families and twins (3) point toward a significant genetic component with a high sibling recurrence risk (4), in only a few cases can a clear genetic component be identified. Various factors – hormonal, immunological, and biochemical – have been implicated in autism’s etiology, but their roles in the symptomology of the disease remain undefined (5). Immune system dysregulation reported in ASD patients includes differential cell fractions and reactivity, autoimmune phenomena, altered cytokine and antibody profiles, and genetic correlations (6). These findings partially corroborate the theory that chronic neurological inflammation in fetal or newborn brain underlies the development of ASD. Subsequent dysregulation of the immune system may contribute to ASD development in genetically susceptible children.

Altered natural killer (NK) cell activity has been reported in the periphery of ASD patients (7). However, these innate immune cells’ function in the pathogenesis of autism is yet unclear. NK cells act specifically against infected or transformed cells, with their actions governed by a balance between activating and inhibitory receptors, each recognizing specific molecules on a given target (8). Killer-cell immunoglobulin-like receptors (KIRs) are a group of cell surface molecules involved in regulating activity of NK (and some T) cells. KIRs are classified as either inhibitory or activating. Their nomenclature is based on the number of extracellular Ig-like domains, and whether the cytoplasmic domain is *long* (L) or *short* (S). The KIR gene family is clustered within the LRC leukocyte receptor complex on chromosome 19q13.4, encoding a total of 14 genes and 2 pseudo genes. KIR genes exhibit allelic and haplotypic variability, while each mature NK cell expresses a specific set of KIRs.

Killer-cell immunoglobulin-like receptor counterparts, the HLA class I ligands, are also extremely polymorphic. This generates an additional level of functional diversity. Both KIRs and their HLA ligands are encoded on different chromosomes (19 and 6, respectively), and therefore segregate independently of each other. This expands the repertoire of possible profiles within any given population (9). Specific KIR–HLA combinations have been suggested as underlying susceptibility as well as resistance to various diseases and conditions (10); the functional implications are less clear. Genetic associations have been reported in infectious diseases (11), autoimmunity (12), inflammatory conditions, reproductive failure, and cancer. Biological models in such studies need to incorporate these receptor:ligand interactions, beyond mere consideration of KIR–HLA genotyping. Indeed, several methods have been proposed to analyze the overall genotyping detected in a specific subject or whole population (13, 14).

**Figure 1:**
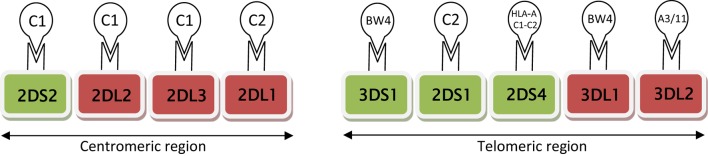
**Schematic diagram of activating (green) and inhibitory (red) receptors on natural killer cells, arranged according to centromic/telomeric location**. These receptors are known to interact specifically with HLA ligands (HLA CI or C2, HLA A and B BW4). HLA-C group 1 (CI) allotypes serve as ligands for KIRs 2DS2, 2DL2, and 2DL3; HLA-C group 2 (C2) functions as ligands for KIRs 2DL1 and 2DS1.

The KIR family predominantly recognizes HLA class I molecules. Specifically, HLA-C is recognized by both inhibitory receptors (KIRs 2DL1, 2DL2, and 2DL3) and activating receptors (KIRs 2DS1, 2DS2, and 2DS4) (Figure 1). HLA-C group 1 (C1) allotypes have an asparagine residue at position 80 (C1:N^80^), and serve as ligands for KIRs 2DL2, 2DL3, and 2DS2. HLA-C group 2 (C2) allotypes function as ligands for KIRs 2DL1 and 2DS1; C2 allotypes have a lysine at position 80 (C2:K^80^). The interactional affinities of these groups may differ. KIR 2DS4 can bind to both HLA-C C1 and C2 allotypes, and certain HLA-A alleles. KIRs 3DL1 and (putatively) 3DS1 interact with HLA-A BW4:I80 as well as HLA-B allotypes [with either isoleucine (BW4:I80) or threonine (BW4:T80) at position 80].

The aim of our research was to genotype the KIR receptors known to interact with HLA ligands in autistic children and their non-autistic parents (mothers and fathers, separately), and to compare KIR receptor: HLA ligand frequencies between these groups. Our hypothesis was that a non-random distribution of these genes profiles should be found if interactions between these two classes of molecules are relevant to autism.

According to the Barker Hypothesis (15), the source of pathologies – including mental disorders such as ASD – can be traced back to embryonic existence in the intrauterine environment (16, 17). KIRs are expressed by maternal NK cells (decidua, uterine lining), which are in close contact with HLA-expressing fetal tissues (placenta). Such prenatal interaction between maternal KIR and the fetal HLA in the uterus may lead to either enhanced NK activation or NK inhibition, which in turn may exert influence upon the placenta and/or developing fetus (18–20). Resulting immune activation and effects upon the central nervous system may lead to neurological and psychiatric disorders in predisposed patients. A mouse model for intrauterine maternal immune activation has already been demonstrated as a possible mechanism for autism- and schizophrenia-related behaviors in offspring (21).

## Materials and Methods

### Participants

This study comprised of 49 ASD-diagnosed children (38 males, 11 females, a male:female ratio of 3.4:1) from Jewish Israeli families, and their parents. Another six sets of parents of ASD-diagnosed children were enrolled, though no DNA samples were available from their children. DNA samples were collected in Israeli special education schools and treatment centers, by the Israeli Society for Autistic Children (ALUT).^1^

Many of the probands had already been diagnosed with ASD by independent clinicians; two trained clinicians confirmed the diagnosis for autism or Pervasive Developmental Disorder-Not Otherwise Specified (PDD-NOS). All probands were diagnosed using the Autism Diagnostic Observation Scale-Generic (ADOS-G) and the Autism Diagnostic Interview-Revised (ADI-R). In addition, parents were interviewed using the Vineland Adaptive Behavior Scales-second edition (Vineland™-II) survey interview edition (VABS).

Probands ranged in age between 25 months to 33 years and 8 months. None of the subjects were known, according to parental reports, to have known genetic factors such as chromosomal aberrations, tuberous sclerosis, or other medical complications that could be related to autism. Parents were screened for psychiatric illness (including ASD); all results were found to be negative.

The Ethics Committee of the Israeli Health Ministry approved this study. All participants signed informed consent forms in advance.

### Genotyping

DNA was extracted from either peripheral blood or buccal cells using a commercial DNA purification kit (Epicentre’s Master Pure™, Madison, WI, USA).

The presence or absence of nine KIR genes known to interact with specific HLA ligands was determined by SSP-PCR (sequence-specific primer polymerase chain reaction), as previously described (22, 23), using nine pairs of primers specific for these KIR genes. This mapping enables verification of presence or absence of a KIR, without discriminating specific heterozygous or homozygote status. A 440/750-bp fragment of the CRP/CAMP, respectively, was included in each PCR as an internal control. The following KIR genes were amplified under identical conditions: 2DL2, 2DL1, 3DL1, 2DS2, 2DS4, and 3DS1. Their PCR thermo-profile comprised 2 min at 92°C; then 25 s at 91°C, 45 s at 65°C, and 45 s at 72°C for 4 cycles; 25 s at 91°C, 45 s at 60°C, and 45 s at 72°C for 26 cycles; 25 s at 91°C, 60 s at 55°C, and 45 s at 72°C for 5 cycles, and 10 min at 72°C (an extension step, in the case of 3DL1 and 3DS1 was set to 2 min). Other KIR amplifications (for 2DL3, 3DL2, and 2DS1) were initiated with denaturation for 2 min at 92°C; then 25 s at 91°C, 45 s at 57°C, and 45 s at 72°C for 4 cycles; 25 s at 91°C, 45 s at 54°C, and 45 s at 72°C for 26 cycles; 25 s at 91°C, 60 s at 50°C, and 45 s at 72°C for 5 cycles, and 10 min at 72°C. All primer sequences, expected PCR products and allelic recoveries are listed in Table 1.

**Table 1:** **KIR genotyping primers**.

KIR	Forward primers (5′–3′)	Amplicon (bp)	Annealing temp (°C)	Control gene	Alleles not detected
	Reverse primers (5′–3′)				
2DS2	CGGGCCCCACGGTTT	240	60	CRP	
	GGTCACTCGAGTTTGACCACTCA				
2DS4	TAGGCTCCCTGCAGTGCG	129	63	CAMP	2DS4*003
	GAGTTTGACCACTCGTAGGGAGC				2DS4*004
					2DS4*006
					2DS4*007
					2DS4*009
2DS1	CTTCTCCATCAGTCGCAT	102	53	CAMP	
	AGGGTCACTGGGAGCTGACAA				
3DS1	GCCCAGCGCTGTGGTGCCTCGC	1958	71	CAMP	3DS1*047
	CTGCAAGGGCACGCATCATGGA				
2DL2	CTGGCCCACCCAGGTCG	173	60	CRP	2DL2*004
	GGACCGATGGAGAAGTTGGCT				
2DL1	AACTTCTCCATCAGTCGCATGAC	100	60	CRP	
	GGTCACTGGGAGCTGACAC				
2DL3	CTTCATCGCTGGTGCTG	516	54	CAMP	2DL3*007
	AGGCTCTTGGTCCATTACAA				
3DL1	CTCCATCGGTCCCATGATGCT	1725	65	CAMP	
	CGCTCTCTCCTGCCTGAACCT				
3DL2	CCCATGAACGTAGGCTCCG	130	54	CRP	
	CACACGCAGGGCAGGG				

*Expected PCR products, annealing temperature (°C), and allelic specificity are given per pair of primers. Primer reference is (22) for all KIRs except 2DS1, for which the primer reference is (23)*.

The distinction of HLA-C KIR ligand groups C1 (N^80^) and C2 (K^80^) was also performed by SSP-PCR. The common HLA-C forward primer was either paired with the C1 (N^80^) or C2 (K^80^) reverse primers, leading to an amplification product of 139 bp (Table 2). This mapping enables discrimination between heterozygous/homozygous status, i.e., C1–C1, C1–C2, or C2–C2. In each HLA PCR reaction, a 440-bp fragment of the CRP gene was included as an internal PCR control. The thermo-profile comprised an initial denaturation of 3 min at 95°C; then 10 s at 95°C, 30 s at 65°C, and 45 s at 72°C for 10 cycles; 10 s at 95°C, 30 s at 58°C, 45 s at 72°C for 22 cycles, and 10 min at 72°C.

**Table 2:** **PCR primers for human leukocyte antigen (HLA) amplification**.

HLA allotype	Direction	Forward primers (5′–3′)	Amplicon (bp)
		Reverse primers (5′–3′)	
HLA-C	Forward	CGCCGCGAGTCCGAGAGG	139
	Reverse (C1: N^80^)	GTTGTAGTAGCCGCGCAGG	
	Reverse (C2: K^80^)	GTTGTAGTAGCCGCGCAGT	
HLA-A BW4:I^80^	Forward	CCATTGGGTGTCGGGTTTC	569
	Reverse	CTCTGGTTGTAGTAGCGGAGCGCG	
HLA-B BW4:I^80^	Forward	ACCCGGACTCAGAATCTCC	366
	Reverse	CTCTGGTTGTAGTAGCGGAGCGCG	
HLA-B BW4:T^80^	Forward	ACCCGGACTCAGAATCTCC	369
	Reverse	CTCTGGTTGTAGTAGCGGAGCAGG	

*Expected PCR product sizes are given. In the case of HLA-C, forward primer was either paired with the C1 (N^80^) or C2 (K^80^) reverse primers, leading to an amplification product of 139 bp*.

*F, forward; R, reverse*.

For HLA-C protocol validation, selected samples (*n* = 30; 10 homozygous C1/C1, 10 homozygous C2/C2 and 10 heterozygous C1/C2) that had previously been typed for HLA-C at high resolution by LIFECODES^®^ HLA SSO Typing Kit (Gen-Probe, Inc.) (Later sequenced by Allele SEQR^®^ HLA PCR/Sequencing Kit, Abbott Molecular) were analyzed by our SSP-PCR.

HLA-A BW4 and HLA-B BW4-I^80^/T^80^ were identified under the same conditions as HLA-C. Validation for HLA-A and B was also performed by selected samples previously genotyped by high resolution methods. All primer sequences [based on Hong et al. (23)] and expected PCR products for HLA-A, -B, and -C genotyping are listed in Table 2.

All amplified SSP-PCR products were visualized by agarose gel electrophoresis using a non-mutagenic fluorescent reagent (“Novel Juice”; GeneDireX, Las Vegas City, NV, USA). Selected sample from each amplified band was sent for sequencing.

### Statistical Analysis

The Wilcoxon Signed-Ranks test (*p* < 0.05) was determined using SPSS software. When combination of more than one factor to specific receptor was detected, some different aspects were detected. The first refers to all ligands equally, and compares the sum of all ligands (relevant to specific receptor) found in each person to determine the mean value. This is defined as “Ligand Composite” (Table 7). Secondly, an alternative designation of “Ligand One+” compares existence of at least one ligand to this specific receptor. This was the case in C1 + 2DL2/2DL3/2DS2, C2 + 2DL1/2DS1 (Table 8). In contrast, for overall signaling (Table 9) and in the case of A_BW4 + 3DL1/3DS1 and B_BW4 + 3DL1/3DS1 (Table 7) only the first option is shown. All HLAB genotyping analysis is described as “total,” meaning having I^80^/T^80^.

## Results

### HLA and KIRs Frequency Comparison

Comparison of the nine different KIR genes and HLA frequencies revealed several main differences between parents of autistic children (Tables 3–5).

**Table 3:** **Inhibitory KIR frequencies in Israeli families with at least 1 ASD-diagnosed child**.

Inhibitory KIR		Child^ASD^	Father	Child^ASD^	Mother	Father	Mother
2DL2	Mean	0.59	0.63	0.58	0.66	0.64	0.68
	*Z*	−0.577		−0.894		−0.408	
	Pearson’s *r*	0.487*		0.204		NR	
2DL1	Mean	0.96	0.98	0.96	0.96	0.98	0.95
	*Z*	−1		0		−1	
	Pearson’s *r*	0.7*		−0.039		NR	
2DL3	Mean	0.86	0.84	0.87	0.89	0.84	0.88
	*Z*	−0.302		−0.333		−0.535	
	Pearson’s *r*	0.135		0.212		NR	
3DL1	Mean	0.94	0.94	0.94	0.89	0.93	0.89
	*Z*	0		−1.342		−0.707	
	Pearson’s *r*	0.645*		0.428*		NR	
3DL2	Mean	1	1	1	1	1	1
	*Z*	0		0		0	
	Pearson’s *r*	NR		NR		NR	

*Z = Wilcoxon Signed-Ranks Test value*.

*NR = not relevant*.

*p ≤ 0.01.

**Table 4:** **Activating KIR frequencies in Israeli families with at least 1 ASD-diagnosed child**.

Activating KIR		Child^ASD^	Father	Child^ASD^	Mother	Father	Mother
2DS2	Mean	0.61	0.63	0.6	0.62	0.64	0.66
	*Z*	−0.302		−0.209		−0.209	
	Pearson’s *r*	0.523**		0.086		NR	
2DS4	Mean	0.96	0.96	0.94	0.89	0.94	0.91
	*Z*	0		−1.342		−0.707	
	Pearson’s *r*	0.478**		0.428**		NR	
2DS1	Mean	0.51	0.37	0.49	0.57	0.34	0.55
	Z	−1.528		−1.069		−2.268*	
	Pearson’s *r*	0.154		0.478**		NR	
3DS1	Mean	0.37	0.39	0.36	0.51	0.39	0.5
	*Z*	−0.728		−2*		−1.279	
	Pearson’s *r*	0.281*		0.419**		NR	

*Z = Wilcoxon Signed-Ranks Test value*.

*NR = not relevant*.

*p ≤ 0.05.

**p ≤ 0.01.

**Table 5:** **HLA ligand frequencies in Israeli families with at least 1 ASD-diagnosed child**.

HLA Ligand		Child^ASD^	Father	ChildASD	Mother	Father	Mother
HLAC1	Mean	0.86	0.9	0.83	0.85	0.88	0.91
	*Z*	−0.707		−0.333		−0.577	
	Pearson’s *r*	0.248		0.371**		NR	
HLAC2	Mean	0.65	0.72	0.68	0.57	0.73	0.52
	*Z*	−0.535		−1.342		−2.058*	
	Pearson’s *r*	0.353**		0.214		NR	
HLA-A-BW4	Mean	0.33	0.37	0.3	0.28	0.36	0.3
	*Z*	−0.577		−0.229		−0.557	
	Pearson’s *r*	0.462**		0.134		NR	
HLA-B-BW4	Mean	0.88	0.86	0.88	0.81	0.87	0.8
	*Z*	0.33		1.272		1	
	Pearson’s *r*	0.203		0.248*		NR	

*Z = Wilcoxon Signed-Ranks Test value*.

*NR = not relevant*.

*p ≤ 0.05.

**p ≤ 0.01.

The most surprising finding is higher frequency of HLA-C2 allotypes in fathers of ASD children (mean 0.73 vs. 0.52, *p* = 0.038), in contrast to C1 frequencies, which were the same in both mothers and fathers (0.88 vs. 0.91) (Table 5). On the other hand, 2DS1, which is known to interact specifically with HLA-C2, was found in higher percentage (0.55 vs. 0.34, *p* = 0.022) in the maternal group (Table 4). Additionally 3DS1, with its specificity against BW4 allotypes, was found to be higher in mothers than in their ASD children (0.51 vs. 0.36, *p* = 0.044) (Table 4). Other KIR frequencies (2DL2, 2DL1, 2DL3, 3DL1, 3DL2, 2DS2, and 2DS4) were similar in both mothers and fathers, and corresponded with values expected in Asian populations.^2^ Most KIR and HLA frequencies in ASD children were similar to those detected in parents.

Positive correlation between parent (mother or father) and child, reflecting the likelihood of transferring the gene (Tables 3–5) were found relative to most of genes, which may reflect a high probability of homozygous genotyping for these genes.

### KIR Signaling Through HLA Molecules

Relevant combinations between KIR receptors and their specific HLA ligands were detected (Table 6). ASD children did not exhibit high frequency of the combination HLA-C2 + KIR2DS1, despite elevated paternal HLA-C2 and maternal 2DS1 frequencies.

**Table 6:** **Frequency of each KIR detected and its corresponding ligand in Israeli ASD families, consisting of both parents and an ASD-diagnosed child**.

KIR		Child^ASD^	Father	Child^ASD^	Mother	Father	Mother
2DL2 + C1	*Z*	−0.832		−1.342		−1.177	
	Pearson’s *r*	0.474**		0.259		NR	
2DL1 + C2	Z	−0.535		−1.091		−1.859	
	Pearson’s *r*	0.372**		0.176		NR	
2DL3 + C1	*Z*	0		0		−0.426	
	Pearson’s *r*	0.227		0.321*		NR	
3DL1 + A_BW4	*Z*	−0.227		−0.243		−0.392	
	Pearson’s *r*	0.148		0.156		NR	
3DL1 + B_BW4	*Z*	−0.302		−1.941*		−1.225	
	Pearson’s *r*	0.283*		0.359**		NR	
2DS2 + C1	*Z*	−0.577		−0.853		−1	
	Pearson’s *r*	0.511**		0.174		NR	
2DS4 + A_BW4	*Z*	−0.577		−0.243		−0.6	
	Pearson’s *r*	0.393**		0.156		NR	
2DS4 + B_BW4	*Z*	−0.302		−1.941*		−1.279	
	Pearson’s *r*	0.211		0.359**		NR	
2DS1 + C2	*Z*	−0.243		−0.775		−0.784	
	Pearson’s *r*	0.132		0.345**		NR	
3DS1 + A_BW4	*Z*	0		−0.577		−0.535	
	Pearson’s *r*	0.456**		0.12		NR	
3DS1 + B_BW4	*Z*	−0.728		−1.414		−0.853	
	Pearson’s *r*	0.25		0.295*		NR	

*Z = Wilcoxon Signed-Ranks Test value*.

*NR, not relevant*.

*p ≤ 0.05.

**p ≤ 0.01.

Correlational analysis between parent (mother or father) and child reflected the likelihood of transferring a gene with its corresponding ligand. Significant values were reported in most cases (Table 6). It is important to mention, these gene families are located on different autosomal chromosomes, supporting homozygous status for most of the genes.

The next evaluation involves the examination of particular HLA ligands with presence/absence of all relevant activating/inhibitory KIR genes (Table 7). “Ligand Composite” analysis (see Materials and Methods) was compared between ASD children and their parents, as well as “Ligand One+” values (Table 8). Significant differences were found between parental groups in the case of C2 + 2DL1/2DS1 “Ligand One+” (*p* = 0.040).

**Table 7:** **Comparison of particular HLA ligands with presence/absence of all relevant activating/inhibitory KIR genes in Israeli ASD families**.

Ligand composite		Child^ASD^	Father	Child^ASD^	Mother	Father	Mother
C1 + 2DL2/2DL3/2DS2	*Z*	−0.363		−0.779		−1.178	
C2 + 2DL1/2DS1	*Z*	−0.213		−0.288		−0.704	
A_BW4 + 3DL1/3DS1	*Z*	−0.216		−0.378		−0.429	
B_BW4 + 3DL1/3DS1	*Z*	−0.382		−0.177		−0.28	

*Z = Wilcoxon Signed-Ranks Test value*.

**Table 8:** **Interactions of KIR and HLA in autistic Israeli patients and their parents, based on overall genotype mapping**.

Ligand one+		Child^ASD^	Father	Child^ASD^	Mother	Father	Mother
C1 + 2DL2/2DL3/2DS2	*Z*	−0.707		−0.333		−0.577	
C2 + 2DL1/2DS1	*Z*	−0.535		−1.342		−2.058*	

*Z = Wilcoxon Signed-Ranks Test value*.

*p ≤ 0.05.

### Overall Balance of Activation and Inhibition

Final analysis (Table 9) presents a full view of the genotyping. It may allow insight to the overall expected interaction of KIR/HLA in Israeli autistic individuals and their non-autistic parents, based on all genotype mapping. Mothers have significantly higher levels of activating signals than their male counterparts (*p* = 0.049).

**Table 9:** **Overall ligand and interaction summaries**.

		Child^ASD^	Father	Child^ASD^	Mother	Father	Mother
KIRs: activating	Mean	2.4043	2.3191	2.3962	2.5849	2.2593	2.6667
	Pearson’s *r*	0.331*		0.274*		0.104	
	*t*-Test	0.443		−1.121		−2.015*	
KIRs: inhibitory	Mean	4.3469	4.3878	4.3585	4.3962	4.4	4.3818
	Pearson’s *r*	0.229		−0.023		0.153	
	*t*-Test	−0.362		−0.286		0.155	
Interactions: activating	Mean	1.9149	1.9787	1.9245	2.0377	1.9259	2.1111
	Pearson’s *r*	0.216		0.142		−0.119	
	*t*-Test	−0.308		−0.636		−0.778	
Interactions: inhibitory	Mean	2.6939	2.7959	2.6981	2.6038	2.8214	2.6607
	Pearson’s *r*	0.272		0.114		−0.099	
	*t*-Test	−0.647		0.582		0.903	

*p ≤ 0.05.

**p ≤ 0.01.

## Discussion

Despite the increased attention garnered by autism research in recent years, the condition is currently diagnosed only after the infant reaches 2 or 3 years of age. Findings in autistic patients indicate multifactorial inheritance, while definitive genetic factors can be diagnosed in only a small percentage of cases. Immunological studies revealed a unique and distinguishing profile in ASD patients compared to controls. In the context of NK cells, altered gene expression, absolute number and function have been reported (7, 24, 25). Reports of modified NK function led us to investigate the highly polymorphic cell surface proteins on NK cells, called KIRs, to search for genetic background underlying the immune abnormalities. By interacting with MHC class I proteins expressed on all cell types, KIRs regulate the killing function of NK.

The most significant finding in this study is the unequal distribution of HLA-C2 (higher in fathers of ASD children) known to interact with KIR 2DS1 (higher in mothers of ASD children) to activate NK function. Analysis of “overall activation” (Table 9) signaling pointed to higher activation in maternal than paternal cohorts. Most HLA and KIR frequencies in ASD children ranged between those of their parents, indicating regular independent genetic inheritance from parents to children. This reinforced the importance of the specific HLA–KIR combinations between parents, though the mechanisms involved are still unclear. Our limitation in interpreting these findings lies in the absence of unusual prevalence of these genes or their combination in the ASD children, in comparison to their healthy parents.

Some reports correlated these initial interactions between parental cells as a possible mechanism for subsequent disease development or other complications. Hiby et al. (26) reported an increased HLA-C2 frequency in different cohorts of affected pregnancies: (a) recurrent miscarriage (RM) couples and their conception products, (b) fetal growth restriction (FGR), and (c) preeclampsia. Conversely, lack of activating KIR (in women affected by RM) or increased inhibitory KIR (in women with preeclampsia) (26, 27) has already been reported. Specifically, KIR 2DS1 seemed to be protective because of its significant lack in RM women. We find this plausible, since activating KIR 2DS1 compete with inhibitory KIR 2DL1 in interaction with HLA-C2, such that a predominance of KIR 2DS1 can overcome the strong inhibitory effect mediated by KIR 2DL1. All these findings support the importance of the innate immune system (mediated by maternal uterine NK and fetal trophoblast cells), modulated by KIR–HLA interaction to play a beneficial role in reproductive success.

Some papers have already pointed out differential segregation of KIR–HLA genes in autistic patients. Our study expands upon this by focusing within ASD families. Torres et al. (28) examined a large autistic cohort, revealing a highly significant increase in the activating KIR gene 2DS1 and its cognate HLA-C2 ligand. This increase in activating KIR may explain, at least partially, the high frequency of autoimmune diseases in autism patients. Guerini et al. (29) compared ASD children to their non-ASD mothers and showed both that activating KIR/HLA combinations were increased and inhibitory KIR/HLA were reduced in ASD children. This difference was proposed as having resulted from the genetic influence of the mothers, as these molecules’ expression and interaction are relevant in the surrounding uterus.

Ethnic populations are known to differ in KIR genotype frequencies and genotype content (30). To the best of our knowledge, this is the first study to research KIR and HLA combinations in the Israeli Jewish population. As shown in the Allele Frequency Net Database,^3^ our geographical location has higher frequency of HLA-C2 (around 0.4–0.7 in Africa and the Middle East). However, we showed here a significant skewing between maternal and paternal C2 frequencies. It raises an interesting question, since HLA clusters map in autosomal chromosomes, leading to independent segregation without respect to gender.

Genotype mapping of this system has certain limitations. While theoretically all gene segments identified by our SSP-PCR system may be expressed on the cell surface (meaning the functional KIR repertoire may depend on the KIR genotype), the transcription and translation processes may lead to different phenotype profiles. Another limitation of the SSP-PCR method in this context is the inability to determine the heterozygous/homozygous status of each of these KIRs. Our future plans are to determine not only genotype, but also RNA and protein levels. Another point to take into account is that NK cell function is a very complicated process determined upon the balance of an array of receptors, of which KIR is only one family. Moreover, the affinity of these interactions may differ (e.g., KIR 2DL3: HLA-C1 interaction is thought to be weak, compared to KIR 2DL2:HLA-C1 or KIR 2DL1:HLA-C2), such that one interaction may wield greater influence than another. It is also becoming increasingly clear that genetic factors, such as promoters and epigenetic mechanisms, are important in controlling NK cell receptor expression and function.

This study comprised a relatively small cohort of ASD-affected families – though statistically verified based on the minor allele frequency (MAF) of C2 (39.5%). In the absence of a control group, our findings were compared with the general population data of our geographical area. Additionally, the clinical patient data have not been thoroughly listed here, places limitations upon the reader in drawing parallels to other patient cohorts. The trends revealed in this preliminary investigation need to be further corroborated in ongoing and expanded research efforts.

Finally, we would like to emphasis another interesting aspect. On the one hand, different pregnancy complications (FGR, spontaneous abortion, placental abruption) are known to stem (at least to some degree) from immunological factors. On the other hand, perinatal factors unrelated to the immunological milieu may also be important in the pathogenesis of ASD. Certain complications during gestation were found to occur with increased frequency in mothers of ASD patients; an immunological basis has been suggested as underlying a portion of these. In some cases, intermediate factors may play a partial role in the association (e.g., reduced birth weight).

## Conclusion

The aim of our research was to genotype the KIR receptors and their relevant HLA ligands in autistic children and their non-autistic parents, in order to screen for possible unique combinations. The most interesting finding in this study is higher frequency of HLA-C2 allotypes in the paternal group besides higher percentage of KIR 2DS1 in the maternal group. Such interaction may lead to NK activation. An absence of unusual prevalence of these genes or their combination in the ASD children, limited our ability to interpret these findings. Moreover, analysis of “overall activation” signaling pointed to higher activation in maternal than paternal cohorts.

## Author Contributions

Mrs. MG is a Ph.D. student. She is the major contributor to this manuscript doing the thinking, bench work, and manuscript writing. Mrs. KA contributed to the bench work as well as writing the manuscript. Prof. NY is an initiator of the original study and has performed the clinical diagnosis of all the participants of the study. Prof. RE is the initiator of original study recruited the families and the original head of the lab. Dr. DM is the head of the lab the study was conducted in, supported the study, and contributed to the manuscript writing and editing.

## Conflict of Interest Statement

The authors declare that the research was conducted in the absence of any commercial or financial relationships that could be construed as a potential conflict of interest.

## Footnotes

^1^http://www.autismaroundtheglobe.org/countries/Israel.asp

^2^http://www.allelefrequencies.net/kir6008a.asp

^3^http://www.allelefrequencies.net/

## Abbreviations

ASD, autism spectrum disorders; HLA, histocompatibility leukocyte antigens; KIR, killer-cell immunoglobulin-like receptor; NK, natural killer.

## References

[1] Caronna EB Milunsky JM Tager-Flusberg H. Autism spectrum disorders: clinical and research frontiers. Arch Dis Child (2008) 93:518–23. 10.1136/adc.2006.115337 18305076

[2] Blumberg SJ Bramlett MD Kogan MD Schieve LA Jones JR Lu MC. Changes in prevalence of parent-reported autism spectrum disorder in school-aged U.S. children: 2007 to 2011-2012. Natl Health Stat Report (2013) 1-11:1following11. 24988818

[3] Persico AM Napolioni V. Autism genetics. Behav Brain Res (2013) 251:95–112. 10.1016/j.bbr.2013.06.012 23769996

[4] Geschwind DH. Genetics of autism spectrum disorders. Trends Cogn Sci (2011) 15:409–16. 10.1016/j.tics.2011.07.003 21855394 PMC3691066

[5] Hu VW. From genes to environment: using integrative genomics to build a “systems-level” understanding of autism spectrum disorders. Child Dev (2013) 84:89–103. 10.1111/j.1467-8624.2012.01759.x 22497667 PMC3402607

[6] Noriega DB Savelkoul HF. Immune dysregulation in autism spectrum disorder. Eur J Pediatr (2014) 173:33–43. 10.1007/s00431-013-2183-4 24297668

[7] Enstrom AM Lit L Onore CE Gregg JP Hansen RL Pessah IN Altered gene expression and function of peripheral blood natural killer cells in children with autism. Brain Behav Immun (2009) 23:124–33. 10.1016/j.bbi.2008.08.001 18762240 PMC2636576

[8] Biassoni R Natural killer cell receptors. Adv Exp Med Biol (2008) 640:35–52. 10.1007/978-0-387-09789-3_4 19065782

[9] Rajalingam R Ashouri E. Gene-specific PCR typing of killer cell immunoglobulin-like receptors. Methods Mol Biol (2013) 1034:239–55. 10.1007/978-1-62703-493-7_12 23775740

[10] Jamil KM Khakoo SI. KIR/HLA interactions and pathogen immunity. J Biomed Biotechnol (2011) 2011:298348. 10.1155/2011/209348 21629750 PMC3100571

[11] De Re V Caggiari L De Zorzi M Toffoli G. KIR molecules: recent patents of interest for the diagnosis and treatment of several autoimmune diseases, chronic inflammation, and B-cell malignancies. Recent Pat DNA Gene Seq (2011) 5:169–74. 10.2174/187221511797636266 21787272

[12] Kulkarni S Martin MP Carrington M. The Yin and Yang of HLA and KIR in human disease. Semin Immunol (2008) 20:343–52. 10.1016/j.smim.2008.06.003 18635379 PMC3501819

[13] Holm SJ Sakuraba K Mallbris L Wolk K Stahle M Sanchez FO. Distinct HLA-C/KIR genotype profile associates with guttate psoriasis. J Invest Dermatol (2005) 125:721–30. 10.1111/j.0022-202X.2005.23879.x 16185272

[14] Zhi D Sun C Sedimbi SK Luo F Shen S Sanjeevi CB. Killer cell immunoglobulin-like receptor along with HLA-C ligand genes are associated with type 1 diabetes in Chinese Han population. Diabetes Metab Res Rev (2011) 27:872–7. 10.1002/dmrr.1264 22069276

[15] Barker DJ Osmond C Golding J Kuh D Wadsworth ME. Growth in utero, blood pressure in childhood and adult life, and mortality from cardiovascular disease. BMJ (1989) 298:564–7. 10.1136/bmj.298.6673.564 2495113 PMC1835925

[16] Gamliel M Ebstein R Yirmiya N Mankuta D. Minor fetal sonographic findings in autism spectrum disorder. Obstet Gynecol Surv (2012) 67:176–86. 10.1097/OGX.0b013e31824bb5d6 22901951

[17] Hagberg H Gressens P Mallard C. Inflammation during fetal and neonatal life: implications for neurologic and neuropsychiatric disease in children and adults. Ann Neurol (2012) 71:444–57. 10.1002/ana.22620 22334391

[18] Bashirova AA Martin MP McVicar DW Carrington M. The killer immunoglobulin-like receptor gene cluster: tuning the genome for defense. Annu Rev Genomics Hum Genet (2006) 7:277–300. 10.1146/annurev.genom.7.080505.115726 16824023

[19] Chazara O Xiong S Moffett A. Maternal KIR and fetal HLA-C: a fine balance. J Leukoc Biol (2011) 90:703–16. 10.1189/jlb.0511227 21873457

[20] Xiong S Sharkey AM Kennedy PR Gardner L Farrell LE Chazara O Maternal uterine NK cell-activating receptor KIR2DS1 enhances placentation. J Clin Invest (2013) 123:4264–72. 10.1172/JCI68991 24091323 PMC4382274

[21] Patterson PH. Immune involvement in schizophrenia and autism: etiology, pathology and animal models. Behav Brain Res (2009) 204:313–21. 10.1016/j.bbr.2008.12.016 19136031

[22] Chainonthee W Bottcher G Gagne K Fussel M Bignon JD Wassmuth R. Improved KIR gene and HLA-C KIR ligand sequence-specific primer polymerase chain reaction genotyping using whole genome amplification. Tissue Antigens (2010) 76:135–43. 10.1111/j.1399-0039.2010.01479.x 20403144

[23] Hong HA Loubser AS de Assis Rosa D Naranbhai V Carr W Paximadis M Killer-cell immunoglobulin-like receptor genotyping and HLA killer-cell immunoglobulin-like receptor-ligand identification by real-time polymerase chain reaction. Tissue Antigens (2011) 78:185–94. 10.1111/j.1399-0039.2011.01749.x 21810083 PMC3150492

[24] Ashwood P Corbett BA Kantor A Schulman H Van de Water J Amaral DG. In search of cellular immunophenotypes in the blood of children with autism. PLoS One (2011) 6:e19299. 10.1371/journal.pone.0019299 21573236 PMC3087757

[25] Vojdani A Mumper E Granpeesheh D Mielke L Traver D Bock K Low natural killer cell cytotoxic activity in autism: the role of glutathione, IL-2 and IL-15. J Neuroimmunol (2008) 205:148–54. 10.1016/j.jneuroim.2008.09.005 18929414

[26] Hiby SE Walker JJ O’Shaughnessy KM Redman CW Carrington M Trowsdale J Combinations of maternal KIR and fetal HLA-C genes influence the risk of preeclampsia and reproductive success. J Exp Med (2004) 200:957–65. 10.1084/jem.20041214 15477349 PMC2211839

[27] Flores AC Marcos CY Paladino N Arruvito L Williams F Middleton D KIR receptors and HLA-C in the maintenance of pregnancy. Tissue Antigens (2007) 69(Suppl 1):112–3. 10.1111/j.1399-0039.2006.762_8.x 17445181

[28] Torres AR Westover JB Givvons C Johnson RC Ward DC. Activating killer-cell immunoglobulin-like receptors (KIR) and their cognate HLA ligands are significantly increased in autism. Brain Behav Immun (2012) 26:1122–7. 10.1016/j.bbi.2012.07.014 22884899 PMC3469320

[29] Guerini FR Bolognesi E Chiappedi M Manca S Ghezzo A Agliardi C Activating KIR molecules and their cognate ligands prevail in children with a diagnosis of ASD and in their mothers. Brain Behav Immun (2014) 36:54–60. 10.1016/j.bbi.2013.10.006 24120931

[30] Takeshita LY Gonzalez-Galarza FF dos Santos EJ Maia MH Rahman MM Zain SM A database for curating the associations between killer cell immunoglobulin-like receptors and diseases in worldwide populations. Database (Oxford) (2013) 2013:bat021. 10.1093/database/bat021 23584834 PMC3625957

